# Optical Quantum Memory and its Applications in Quantum Communication Systems

**DOI:** 10.6028/jres.125.002

**Published:** 2020-01-16

**Authors:** Lijun Ma, Oliver Slattery, Xiao Tang

**Affiliations:** 1 National Institute of Standards and Technology,Gaithersburg, MD 20899 USA

**Keywords:** quantum communication, quantum key distribution, quantum memory, quantum network, quantum repeater

## Abstract

Optical quantum memory is a device that can store the quantum state of photons and retrieve it on demand and with high fidelity. It is emerging as an essential device to enhance security, speed, scalability, and performance of many quantum systems used in communications, computing, metrology, and more. In this paper, we will specifically consider the impact of optical quantum memory on quantum communications systems. Following a general overview of the theoretical and experimental research progress in optical quantum memory, we will outline its role in quantum communications, including as a photon source, photon interference, quantum key distribution (QKD), quantum teleportation, quantum repeater, and quantum networks.

## 1 Introduction

Quantum communication is a technology that uses the quantum properties of information carriers, such as single photons, to realize quantum information exchange between parties. The technology has many unique applications that are impossible in classical communication systems. Currently, there are two main applications of quantum communication: quantum key distribution (QKD) and quantum entanglement distribution.

QKD provides secure encryption keys over unsecured communication channels. Its security is guaranteed by the fundamental quantum properties of single photons instead of mathematical complexity. Charles Bennett and Gilles Brassard proposed the first QKD protocol in 1984 [1], which was later given the name of BB84. The first demonstration of a QKD system was completed in 1989, in which the quantum channel was based on a 30 cm long free-space path located inside their laboratory. Since then, QKD technology has matured into one of the major schemes in cryptography applications.

Quantum entanglement distribution over long distances and over large numbers of quantum memories is vital to implement a quantum computational network and to realize large-scale quantum computation and processing. Quantum entanglement enables quantum teleportation–based communication, which, for long distances, requires quantum repeaters to overcome the exponential scaling of loss with distance in an optical fiber. One paradigm for implementing quantum repeaters is the distribution of entanglement between remote quantum memories.

However, QKD and quantum entanglement distribution still face some significant technical challenges. Although, in principle, QKD promises unconditional security in data communication, in practice, there are many security issues caused by imperfect devices. For example, since it is very difficult to get a true single photon source, such QKD protocols usually use a weak coherent laser beam as a source. However, the coherence state includes multiphoton components, which make the system susceptible to a photon number splitting (PNS) attack [2]. In a PNS attack, an eavesdropper, usually called Eve, is able to obtain information about the key without being traced. To address the security issue caused by PNS attack, decoy QKD was proposed by Hwang in 2003 [3]. Decoy QKD uses multiple intensity levels at the transmitter’s source and monitors the error rates associated with each intensity level to detect PNS. In addition, the measurement devices are still prone to other forms of attacks by Eve, such as the time-shift attack [4], phase-remapping attack [5], and blinding attack [6]. These attacks have been demonstrated experimentally on QKD commercial products [6, 7]. To solve these implementation security challenges, measurement device independent QKD (MDI-QKD) was proposed [8]. MDI-QKD makes the QKD system immune to all kinds of hacking attempts of the measurement device, including single photon detectors. However, the MDI-QKD protocol needs to conduct a Bell state measurement (BSM) from two independent single photon sources, and a successful BSM needs both photons to successfully arrive at the interference point within the coherence time of the photons. This requirement greatly reduces the key rate that can be generated in the system. Quantum entanglement distribution also faces significant technical challenges, and although quantum teleportation has been demonstrated by many groups, a fully functioning quantum repeater has not been realized.

Quantum memory can temporarily store a quantum state, and then retrieve it on demand later and thereby help to solve some critical technical issues and enhance the quantum communication system performance. In this paper, we provide a general overview of the theoretical and experimental results of optical quantum memory research and discuss its applications in quantum communication systems to date. In Sec. 2, cavity-based (Sec. 2.1) and storage medium–based (Sec. 2.2) quantum memory schemes are introduced. In Sec. 3, the applications of quantum memories in single photon sources (Sec. 3.1), interfaces (Sec. 3.2), MDI-QKD (Sec. 3.3 and Sec. 3.4), teleportation (Sec. 3.5), quantum repeaters (Sec. 3.6). and the quantum internet (Sec. 3.7) are described.

## 2 Optical Quantum Memory

Although a qubit can be realized in many different physical systems, such as atoms, ions, and superconducting circuits, the most prominent physical realization of a qubit in quantum communication is based on photons. The qubit information can be encoded on photonic quantum states as polarization, phase, and orbital angular momentum states. Furthermore, the photon rarely interacts with the environment and is also compatible with current optical networks, so it is the best candidate for free-space and fiber-based quantum communication. The quantum memory used with photonic qubits, in which the quantum states are prepared and manipulated using photons, is called optical quantum memory. Optical quantum memory is typically based on single photon storage, and two single photon storage devices in an interferometrically stable system can store the quantum state of an encoded photon. Optical quantum memories have been actively pursued for several years, and various approaches have been proposed and experimentally demonstrated [9-16].

### 2.1 Cavity-Based Quantum Memory

**Fig. 1:**
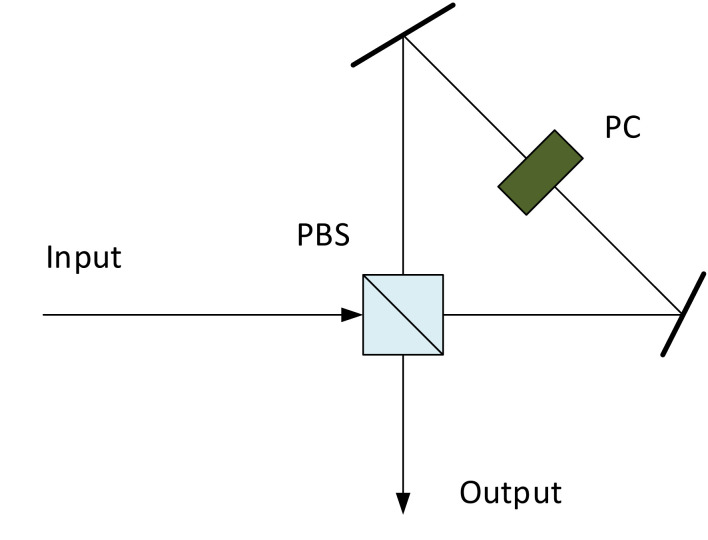
Schematic structure of cavity-based quantum memory. PBS: polarizing beam splitter; PC: Pockels cell.

One relatively straightforward way for implementing quantum memory is by using a cavity to retain the photon and release it when needed. Figure 1 shows the structure of a cavity-based quantum memory consisting of a polarizing beam splitter (PBS), a Pockels cell (PC), and two mirrors. After a photon enters the cavity, it can be trapped inside by a polarization setting of the PC and released by changing the setting of the PC. The main advantages of a cavity-based quantum memory are its simple and inexpensive configuration and the very broad working wavelength range. However, due to the loss of the cavity (caused by the imperfect polarization extinction of PBS, the insertion loss of the PC, and the imperfect reflection of mirrors), the cavity cannot provide a long storage time. In addition, the storage pulse duration is usually limited by the cavity length. The cavity-based quantum memory has been demonstrated to create a pseudo-deterministic photon source, to create various multiphoton state systems [17], and to enhance the photon interference efficiency for quantum communication [18].

### 2.2 Media-Based Quantum Memory

Currently, most approaches to quantum memories are based on storage media, such as atoms, atomic ensembles, ions, or molecules. In these approaches, the quantum state of the photon (called a flying qubit) is converted to the quantum state of the storage media (called a stationary qubit). After the required storage time, the stationary qubit is converted back into a flying qubit. Currently, the media-based quantum memory approaches can be categorized into three schemes: optically controlled, engineered absorption, and a hybrid of these two approaches [19].

#### 2.2.1 Optically Controlled Quantum Memory

**Fig. 2:**
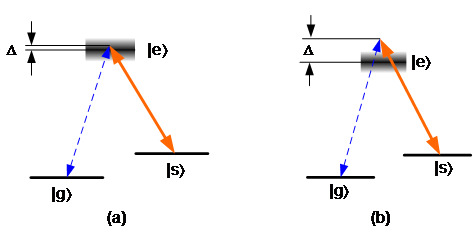
Optically controlled scheme: (a) energy-level structure of EIT approach, and (b) energy-level structure of Raman approach.

In the optically controlled scheme, a strong optical pulse is used to induce the absorption of photons into the storage medium. Currently, the two main approaches in this scheme are called electromagnetically induced transparency (EIT) and Raman quantum memory, respectively. The two approaches were first proposed in 2000: the EIT approach by Fleischhauer and Lukin [19], and the Raman approach by Kozhekin, Mølmer, and Polzik [20]. Both EIT and Raman use a Λ (lambda)–type three-energy-level structure, as shown in Fig. 2. The transitions between the ground levels (|g〉 and |s〉) and the excited level (|e〉) are allowed and used for the signal and the control light. The transition between two ground levels meanwhile is electric dipole forbidden, which enables a long storage time. The storage and retrieval processes of both EIT and Raman approaches are similar. A strong control beam, the wavelength of which is close to resonance between the |s〉 and |e〉 levels, is first applied to the storage medium and prepares all atoms in the ground level. The single photon signal beam, the wavelength of which is close to the resonance between |g〉 and |e〉, carries the quantum information and copropagates with the control beam into the storage media. The control and signal fields are in a two-photon resonance condition with a certain amount of frequency detuning (denoted as ∆) from the excited level (|e〉). When the signal pulse fully enters the storage media, the intensity of the control beam is reduced adiabatically to zero. The quantum state of the signal photon is coherently mapped onto and stored as an atomic spin wave. After a certain storage time, the signal photon with the stored quantum state is retrieved by reapplying the control beam. One advantage of this scheme is that it is retrievable on-demand: The signal photons are emitted immediately once the control beam is reapplied. The main difference between the EIT and Raman approaches is the frequency detuning, ∆. In EIT quantum memory, the ∆ is small, so as to maintain the resonant condition. As a result, the power of the control beam can be efficient, but its usable bandwidth is typically limited to sub-megahertz (warm atom) or tens of megahertz (cold atom) levels. In the Raman approach, the ∆ is much larger because it uses an off-resonant Raman interaction. The main advantage of the Raman approach is its wider operating bandwidth, and storage bandwidths of gigahertz using cesium (Cs) and rubidium (Rb) atoms and terahertz using diamond and hydrogen molecules have been demonstrated. Therefore, Raman quantum memory can store a very short signal pulse that consists of a large range of frequencies and is suitable for high-speed quantum communication systems.

The main challenge of optically controlled quantum memories is the noise, in particular, as the single photon level signal is emitted with a strong control beam. In this case, the residual control beam becomes a serious source of noise in the single photon signal band. The noise issue is particularly severe in the Raman quantum memory, since it works at an off-resonance range and requires a very strong control beam. Additionally, spontaneous four-wave mixing (SFWM) is likely to be caused by the strong control beam and thus results in noise photons that are in the same spatial, temporal, and frequency mode as the retrieved signal photons. Recently, Raman quantum memory based on cold atoms that can use a smaller off-resonant detuning and a weaker control beam has been demonstrated with very low noise and used for nonclassical light storage. Both EIT and Raman approaches have been demonstrated in many storage media, including single atoms, warm atomic vapors, laser-trapped cold atoms, Bose-Einstein condensates (BEC), and rare-earth–doped solid state materials.

#### 2.2.2 Engineered Absorption Quantum Memory

Another important scheme of quantum memory is called engineered absorption, which is based on the photon echo effect [21]. There are two main approaches in this scheme: controlled reversible inhomogeneous broadening (CRIB) and atomic frequency combs (AFC).

**Fig. 3:**
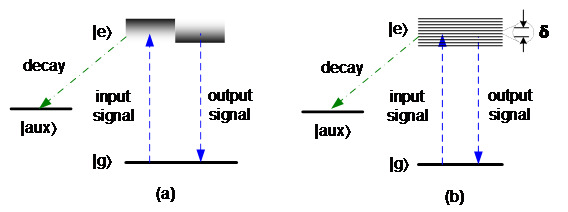
Engineered absorption schemes: (a) energy-level structure of CRIB, and (b) storage and retrieval process of CRIB.

The CRIB approach was first proposed in 2001 by Moiseev and Kröll [22]. In this approach, the storage atoms are first excited by an optical pump with a spectrally burned hole. The pump creates a narrow absorption line in the atoms and transfers other atom populations into an auxiliary state. The narrow absorption line is inhomogeneously broadened by an external electric or magnetic field to store the input signal photons. After a certain time, the external electric or magnetic field is reversed, and the stored signal photons are re-emitted by photon-echo. The energy-level structure of CRIB is shown in Fig. 3(a). It is called transverse CRIB if the direction of variation of the external electric or magnetic field is perpendicular to the propagation of the signal photons or longitudinal CRIB (also called gradient echo memory [GEM]) if the two directions are parallel. In GEM, the atomic detuning has a linearly varying spatial dependence, which allows for a unity efficiency without additional techniques such as cavities or phase-matching optical fields.

The AFC approach was first proposed in 2009 by Afzelius et al. [23]. Instead of using an optical pump with a spectrally burned hole, AFC uses a spectrally comb-structured optical pump. The broad optical transition is shaped into periodic and narrow absorption lines equally spaced in the frequency domain by the optical pump. The input photon signal usually covers a few lines of the comb. After absorption of a photon signal, the state of the photon is transferred to the collective atomic excitation, and atoms at different frequencies begin to dephase. Because of the periodic structure of the comb, a rephasing process occurs after a certain time that corresponds to the inverse of the frequency comb period (δ). When all the atoms return to an in-phase status, the photon with the same quantum state is re-emitted in the forward direction. The energy-level structure of AFC is shown in Fig. 3(b). The AFC approach requires two energy levels for the storage and retrieval process, and an auxiliary level for the absorption line structuring. However, AFC approach does not need a reverse process to retrieve the photons, because the rephasing and re-emission of photons occurs after a certain time predetermined by the period of the absorption comb lines (1/δ). The advantage of the AFC approach is its multimode capacity, which is given by the number of AFC comb lines and is fully independent of the medium optical density. In contrast, the multimode capacity of CRIB scales linearly with optical depth, while that of the EIT and Raman approaches scales with the square root of the optical depth [24].

Currently, most CRIB and AFC quantum memory schemes use rare-earth–doped crystals at cryogenic temperatures (<4 K) and have been demonstrated in a variety of operating wavelengths, including the telecom wavelength with erbium-doped crystals [25] and erbium-doped fiber [26]. Some GEM approaches have also been demonstrated with a warm Rb atomic vapor [27, 28].

#### 2.2.3 Hybrid Quantum Memory

In a hybrid scheme that combines these two approaches, the storage medium is similar to the engineered absorption scheme, and it also uses a control beam and three-energy-level structure like the optically controlled scheme. Therefore, the hybrid scheme retains most of the advantages of both the optically controlled and engineered absorption schemes, such as long storage time, low noise, high mode capacity, and on-demand readout (although it has a delay after the second control pulse). So far, two hybrid approaches, called Raman-GEM [29] and Λ-AFC [30, 31], have been successful demonstrated.

**Fig. 4:**
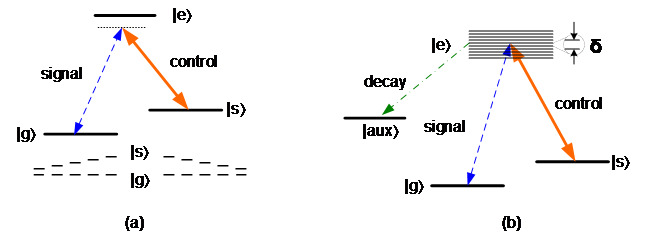
Hybrid scheme: (a) energy-level structure of Raman-GEM, and (b) storage and retrieval process of Raman-GEM.

Raman-GEM, for which the energy structure is shown in Fig. 4(a), combines the Raman and GEM approaches. This approach does not need initial preparation of an absorption line and uses the ground-state spin transition. The transition lines are gradient longitudinally broadened by an external magnetic field. A strong control beam maps the signal state onto the broadened line coherently. During the retrieval process, photons are re-emitted after reversing the polarity of the magnetic field and re-applying the control pulse. This approach has demonstrated a very high storage efficiency of 87% [29].

Λ-AFC is another quantum memory hybrid approach. It uses a three-energy-level structure and an auxiliary level for initialization, as shown in Fig. 4(b). In Λ-AFC, the broadened transition is shaped into an atomic frequency comb by using a spectrally comb-structured optical pump, just like the two-level AFC. When a photon signal comes in, a strong control beam maps the coherence onto the spin wave of the atoms. During the retrieval process, another control beam is applied, and the process for the rephasing resumes, and the photon is re-emitted. In addition to a fixed rephasing time (1/δ), Λ-AFC has an additional spin wave storage time (Ts). Therefore, Λ-AFC has a much longer storage time than the AFC approach. Λ-AFC also keeps the high multimode capacity of AFC. In comparison to Raman-GEM, Λ-AFC has the advantage of the storage time being the same for all photons, and thus the order of output photons is the same as the input photons.

Because most of the media-based approaches use atomic ensembles, the operating wavelength is usually the transition wavelength of atoms, and the working bandwidth is limited. The simplest way to get photons compatible with atomic-based quantum memory is to generate the photons from a system of the same atoms. However, the wavelength and bandwidth may not compatible with the telecom wavelength and other photon sources and devices in a quantum network. To integrate the quantum memories into a quantum network, another two technologies are also important: (1) a quantum interface based on single photon frequency conversion that can convert the photons’ wavelength between the memory and telecommunications wavelengths [32], and (2) a narrow bandwidth photon source based on, for example, cavity-enhanced technology that ensures the wavelength and bandwidth of photons are compatible with quantum memories [33].

## 3 Quantum Communication System with Quantum Memories

### 3.1 Memory-Assisted Photon Source

**Fig. 5:**
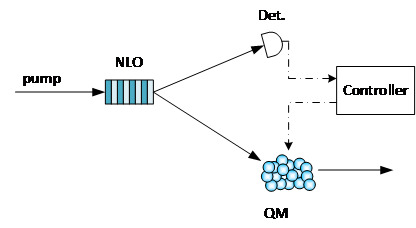
Memory-assisted photon source. NLO: Nonlinear Optics Crystal; QM: Quantum Memory

Photon sources are one of most important devices for any quantum communication systems. A deterministic photon source, which can emit a single photon at any time defined by the user, is very useful for a quantum communication system. Currently, most entangled photon sources are based on spontaneous processes, such as spontaneous parametric down conversion (SPDC) or spontaneous four-wave mixing (SFWM). In such photon sources, the creation of photons is probabilistic. As the photons are created in pairs, one photon can be used to herald the creation of the other. With a quantum memory (QM), a herald photon source can be converted to a deterministic photon source [17]. As shown in Fig. 5, two photons are created in a pair, and one photon (the heralding photon) is sent to a detector, and the other (the heralded photon) is sent to a quantum memory. Once the heralding photon is detected, a heralded photon is stored in the quantum memory and can be emitted as needed.

### 3.2 Memory-Assisted Photon Interference

**Fig. 6:**
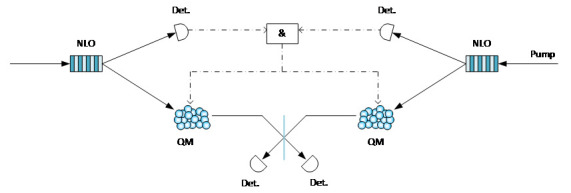
Memory-assisted photon interference.

The two-photon interference effect in quantum optics was first demonstrated by C. Hong, Z. Ou, and L. Mandel in 1987, and it is known as the Hong-Ou-Mandel (HOM) effect. The effect, which is the experimental basis for the Bell state measurement (BSM) of photons, states that two indistinguishable photons can interfere on a beam-splitter even if the two photons come from different sources. Many quantum communication systems and processes, such as MDI-QKD, quantum teleportation, and quantum repeaters need to achieve independently sourced photon interference. However, this process requires that the two photons from different sources arrive at the beam-splitter at the same time. The requirement greatly reduces the success rate of photon interference when low-efficiency deterministic sources or probabilistic sources are used. Two quantum memories, on the other hand, can be used to store the two photons independently even if generated at different times and can then emit them at the same time to achieve higher photon interference success rates. With quantum memory, the photon interference efficiency will be significantly enhanced [18]. Figure 6 shows a setup for memory-assisted photon interference. Two independent probabilistic photon pair sources generate two pairs of photons. One photon from each pair is detected for heralding, while the other from each pair is stored in quantum memory. When heralding signals indicate that both memories have photons ready, the two stored photons are emitted simultaneously for photon interference.

### 3.3 Memory-Assisted MDI-QKD

**Fig. 7:**
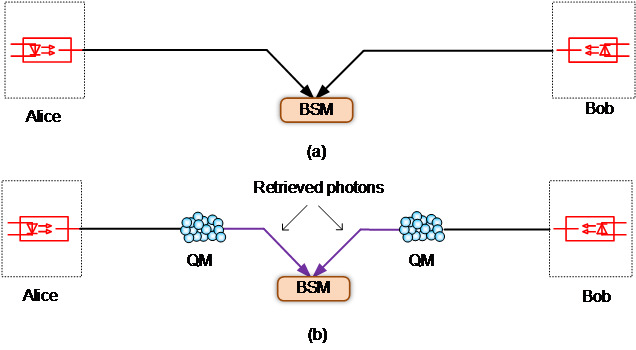
(a) Original MDI-QKD and (b) memory-assisted MDI-QKD.

As we discussed in the introduction section, traditional QKD systems suffer from many attacks on their imperfect measurement devices. MDI-QKD was proposed to improve the security of practical QKD systems with imperfect measurement devices. The MDI-QKD has proven to be more tolerant to single photon detector imperfections, such as low detection efficiency, dark counts, and dead time. In the original MDI-QKD protocol, shown in Fig. 7(a), two ends called Alice and Bob, respectively, send encoded photons to a BSM site. After the BSM bases are announced publicly, Alice and Bob postselect their results by discarding the ones that do not match their measurement bases. Then, they use the remainder for key generation after error correction and privacy amplification. The measurement in this protocol is done by an untrusted party, and therefore MDI-QKD is resilient to detector attacks.

However, the original MDI-QKD protocol needs to conduct the BSM from two independent single photon sources, and a successful BSM needs both photons in the same cycle to successfully arrive at the interference point within the coherence time of the photons. Therefore, the key generation rate inversely scales with the loss throughout all the links. Memory-assisted MDI-QKD, shown in Fig. 7(b), was proposed to enhance the key rate by equipping both sides of the BSM with quantum memories [34]. The arriving photons are stored in the quantum memories first, and then they are released at the optimal time into the BSM. This protocol does not require both photons to be generated at same time, since they can be synchronized by the inserted quantum memories. In this case, the gated photon detectors at the end of a BSM can also be precisely synchronized, and the influence of the detection dark counts can be greatly reduced. Therefore, the BSM efficiency of two retrieved photons can be much higher, and the entire system key rate can be greatly increased.

### 3.4 Towards a Long-Distance MDI-QKD System

Quantum repeaters are considered the most viable solution to enable secure quantum communication for long-distance operation, yet they face many technical challenges, and there has been no fully operational quantum repeater developed to date. However, some new protocols based on memory-assisted MDI-QKD can offer a midterm solution for longer-distance QKD systems, even though they are not scalable as quantum repeaters.

**Fig. 8:**
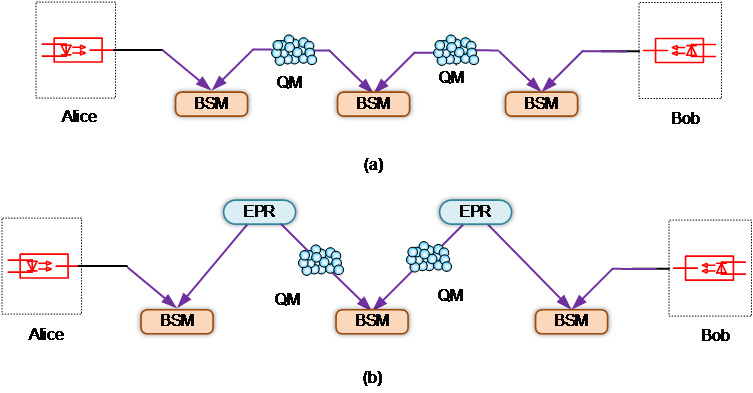
(a) Entangled memory-assisted MDI-QKD and (b) entangled memory-assisted MDI-QKD with entangled photon sources. EPR: Entangled Photon Source; BSM: Bell State Measurement.

Entangled memory-assisted MDI-QKD is one such protocol [35-37]. Instead of using quantum memory to store the photons that are transmitted by Alice and Bob, this protocol uses a quantum memory–based photon source to generate entangled photon pairs that in turn become entangled with incoming photons in a BSM. As shown in Fig. 8(a), two quantum memories first implement an entangling process. Each of the two quantum memories generate entangled photon pairs and send one of their photons to interfere with photons from Alice or Bob in separate BSMs. Once these BSMs succeed, each quantum memory is entangled with the photon coming from Alice or Bob. After both quantum memories complete the entangling process, they send out their second stored photon for another BSM operation.

The entangled memory-assisted MDI-QKD can also use external entangled photon sources to further enhance efficiency [35-37]. As shown in Fig. 8(b), two photon sources emit entangled photon pairs. One photon is stored in a quantum memory, while the other photon is sent to a BSM for interference with photons from Alice or Bob to obtain BSM results. Once both sides of the BSM are successful, the other stored photon can be emitted from quantum memory and sent to perform a BSM as a part of the original memory-assisted MDI-QKD protocol. This protocol could provide a longer transmission distance and faster rates and avoid the multiphoton excitation effect to which ensemble-based quantum memories are prone.

### 3.5 Quantum Teleportation with Quantum Memory

**Fig. 9:**
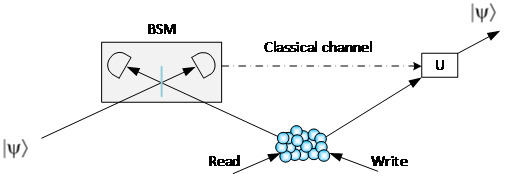
Quantum teleportation with quantum memories. U: Unitary operator.

Quantum teleportation is a way to transfer a quantum state from one location to another and is the basis of quantum repeaters and quantum networks. Quantum teleportation was initially proposed in 1993 by Bennett *et al*. [38]. The basic protocol of quantum teleportation is (1) an entangled photon source (EPR photon source) generates a pair of entangled photons; (2) one photon of the pair is sent to interfere with an incoming photon with an unknown quantum state to implement a BSM; and (3) the unknown quantum state is projected onto the other photon according to the BSM result. Since the first experiment of quantum teleportation was realized in 1997 [39], many experiments have been successfully demonstrated, including a 1400 km teleportation between a satellite and the ground [40]. Although teleportation itself does not need quantum memory, a combination of quantum teleportation and quantum memory offers many advantages, including, for example, retrieving a teleported photon at a suitable time and making teleportation scalable. Reference [41] demonstrated quantum teleportation with quantum memories. As shown in Fig. 9, the experimental setup includes a memory-based photon source that is triggered by a write pulse and sends a photon to interfere with an incoming unknown photon. Once the BSM is completed, and the result is transmitted over a classical channel, the stored photon can be emitted when needed. The unknown quantum state is projected onto it by applying an adjustment according to the BSM result.

### 3.6 Quantum Repeater

The main challenge that long-distance quantum communication systems and quantum networks face is the fiber attenuation and protocol operations errors. To overcome the losses and errors during the photon transmission, a quantum repeater was proposed [42, 43]. A quantum repeater has two main functions: (1) compensate the transmission loss, and (2) correct the operation errors. According to the ways in which the two functions are realized, quantum repeaters are categorized into three so-called generations [44, 45]. The first-generation quantum repeater uses heralded entanglement generation (HEG) to compensate for the losses and heralded entanglement purification (HEP) to correct the operation errors. Because HEP requires two-way classical communication between repeater stations to confirm the success of purification, the first-generation quantum repeater requires quantum memory with a storage time that is longer than the time required for the two-way classical communication. The second-generation quantum repeater still uses HEG to compensate for the losses, but it uses quantum error correction (QEC) to correct the operation errors. QEC requires one-way communication, and this greatly reduces the quantum memory storage time requirement. The third-generation quantum repeater uses QEC for both functions. In a certain scenario, the third-generation quantum repeater does not require quantum memory.

In comparison to HEG and HEP, QEC is technically more difficult. QEC requires high-fidelity quantum gates for which the operation errors are below the fault tolerance threshold, a target that is far away from being implemented. Although the first-generation quantum repeater with HEG and HEP is also challenging, some practical protocols have been proposed, and some proof-of-principle experiments have been implemented.

**Fig. 10:**
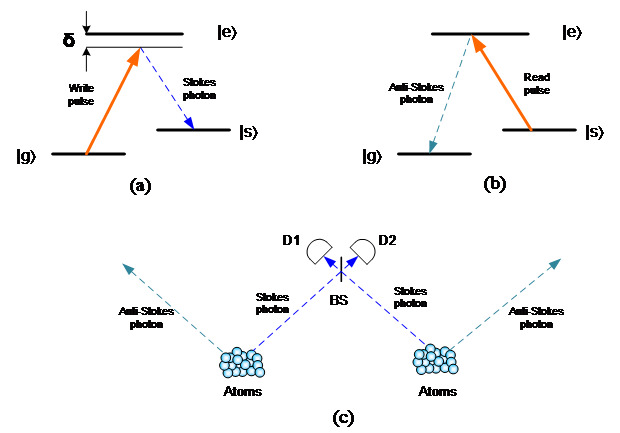
DLCZ protocol. BS: beam splitter, D1 and D2: single photon detector.

The first quantum repeater protocol was proposed by Duan, Lukin, Cirac, and Zoller in 2001, and it is known as the DLCZ protocol [46]. In the DLCZ protocol, quantum memories are entangled, and the quantum states are transferred between the quantum memories and photons. The DLCZ protocol is based on atomic ensembles with a three-energy-level (Λ) configuration. As shown in Fig. 10, the protocol first prepares all atoms in each of the memories in the ground state. For the write process of the protocol, a weak write pulse creates an atomic excitation that induces a stokes photon from the quantum memory. When the two memories are simultaneously illuminated by write pulses, and the emission paths of the emitted photons are combined to interfere at a beam splitter, then the result will be a single click at one of the detectors. Since it is impossible to tell which one of the two memories emitted the photon, the state of the two memories becomes an entangled superposition. At a later time, a relatively strong read pulse is used to convert the stored atomic excitation into an anti-Stokes idler photon. This is a measurement-induced protocol, and the entanglement of memories can be transferred to photonic modes on demand and can be subsequently used as the basis of a quantum repeater. The DLCZ protocol can reduce the fiber distance loss from the exponential scale (~O(*e^L^*)) to the polynomial scale (~O(*L*)), where *L* is the transmission distance [43].

Since the DLCZ protocol was first proposed, several improvements have been made, such as, protocols based on entanglement swapping via two-photon detection [47, 48], protocols on entanglement generation via two-photon detection [49], protocols based on single photon sources [50], and protocols based on entangled photon sources and quantum memories [51, 52]. Among these protocols, those based on entangled photon sources and quantum memories can use temporal multiplexing [51] or spatial multiplexing [52]. The multiplexing technology can enhance the rate of quantum repeaters and extend the communication distance, especially by combining the temporal and spatial multiplexing in the same system [43]. Multimode quantum memory is needed for temporal multiplexing. Among all current quantum memory approaches, AFC-type quantum memories have the best multimode capacity and are promising for such temporal multiplexing protocols.

Quantum memory is the key device for DLCZ protocol and its variations. However, currently, the performance of the quantum memories, especially the limited coherent time, cannot fully satisfy the requirements of quantum repeaters, and this remains the major obstacle to realize a practical quantum repeater. A new protocol for a quantum repeater without quantum memory has recently been proposed, called an all-photonic quantum repeater [53]. The all-photonic quantum repeater uses repeater graph states (RGS) to eliminate the need for quantum memories. However, it requires many multiple entangled photon sources and photon interferometers, which is technically very challenging. To date, only a 2 × 2 parallel all-photonic quantum repeater has been demonstrated [54]. It is believed that some combination of the all-photonic protocols and the quantum memory–based protocols could one day enable a practical quantum repeater. This hope is based on the fact that RGS can relax the requirement for the coherence time of quantum memory, while a quantum memory can reduce the size requirement for the RGS.

### 3.7 Towards Quantum Internet

Quantum communication is not limited to point-to-point systems. A quantum network can connect quantum computers at different locations to achieve unparalleled capabilities that are impossible for classical systems. Large-area quantum networks with worldwide quantum connections, *i.e*., the quantum internet [55], will have a revolutionary impact on communication, computation, and measurement.

In a scalable quantum network, these quantum memory nodes will be required to store quantum information, convert the quantum information into flying qubits, and send the quantum information into the network interconnects on demand. In addition, quantum memories will play an important role in quantum repeaters and quantum computers. Therefore, quantum memories with high efficiency, high fidelity, and long storage time will be an indispensable technology for the future quantum internet.

**Fig. 11:**
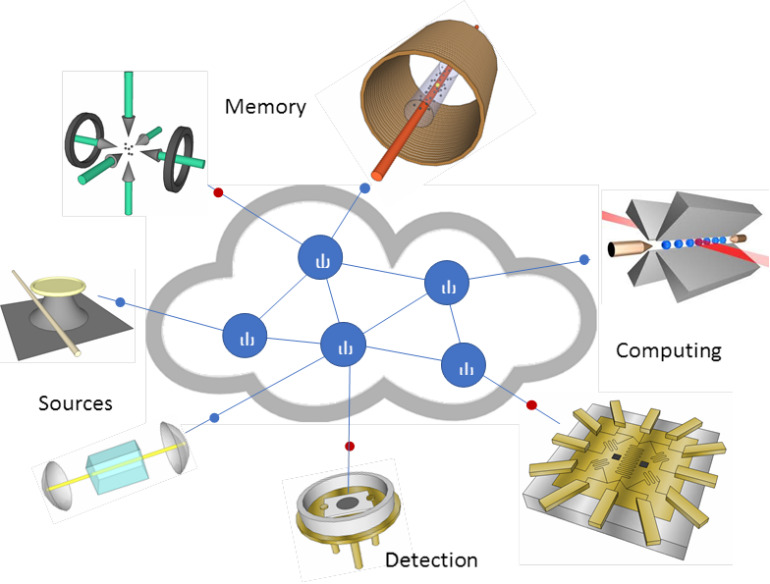
Quantum internet.

## 4 Conclusion

Optical quantum memory is a device that can store the quantum state of photons and retrieve it with high fidelity on demand. This review provided a general overview of the principles and the main experimental results of optical quantum memory, including current approaches such as cavity-based memory, EIT, Raman, CRIB, and AFC. We also discussed its applications in quantum communication components and systems, including single photon sources, QKD systems, quantum teleportation, quantum repeaters, and future quantum networks. Quantum memory is an essential building block in quantum communications and networks and will play an ever more important role in future quantum information technology and quantum computation research and development.
